# Library Preparation for Genome-Wide DNA Methylation Profiling

**DOI:** 10.21769/BioProtoc.5488

**Published:** 2025-11-05

**Authors:** Fei-Man Hsu, Matteo Pellegrini, Pao-Yang Chen

**Affiliations:** 1 Department of Molecular, Cell and Developmental Biology, University of California Los Angeles, CA, USA; 2 Institute of Plant and Microbial Biology, Academia Sinica, Taipei, Taiwan

**Keywords:** DNA methylation, Whole genome bisulfite sequencing, Epigenetics, Epigenomics, Library preparation, Next generation sequencing

## Abstract

DNA methylation is a fundamental epigenetic mark with critical roles in epigenetic regulation, development, and genome stability across diverse organisms. Whole genome bisulfite sequencing (WGBS) enables single-base resolution mapping of cytosine methylation patterns and has become a standard method in epigenomics. This protocol provides a detailed, step-by-step workflow for WGBS library construction starting from genomic DNA. It includes steps of RNaseA treatment, DNA shearing, end-repair and A-tailing, adapter ligation, bisulfite conversion, library amplification, and quantification. Notably, the method uses self-prepared reagents and customizable index systems, avoiding the constraints of commercial library preparation kits. This flexibility supports cost-effective, scalable methylome profiling, suitable for diverse experimental designs, including high-throughput multiplexed sequencing.

Key features

• Provides a comprehensive workflow for whole-genome bisulfite sequencing (WGBS) library preparation without relying on commercial kits.

• Enables flexible multiplexing by allowing users to customize index systems during oligonucleotide synthesis.

• Offers high-resolution, unbiased DNA methylation profiling at single-base resolution.

• Optimized for cost-effective and scalable applications, including high-throughput sample processing.

• Compatible with a wide range of DNA inputs and adaptable to different species and experimental designs.

## Graphical overview


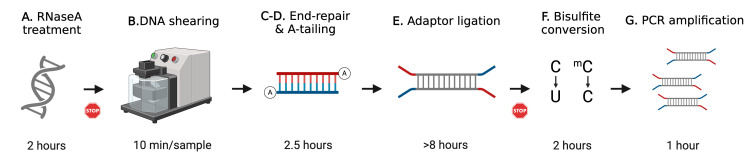


**Schematic overview of whole genome bisulfite sequencing (WGBS) library preparation.** This protocol describes step-by-step WGBS library preparation. Starting from genomic DNA (gDNA), RNaseA treatment removes excessive RNA. gDNA is then fragmented by ultrasonication. End-repair and A-tailing allow DNA fragment ends to be modified for adapter ligation. After a few cycles of PCR amplification, the final WGBS library is then quantified and verified for concentration and size distribution. The STOP signs represent safe stop points.

## Background

DNA methylation is one of the most studied epigenetic modifications, involving the addition of a methyl group to the fifth carbon of a cytosine (5C), resulting in 5-methylcytosine (5mC). Under high temperature for a period, the double-stranded DNA is denatured, sodium bisulfite is able to react with the unmethylated cytosines, and a sulfonated cytosine intermediate is formed. This intermediate is then converted through hydrolytic deamination, followed by an alkaline treatment, and the cytosine is fully converted to uracil (U). This process, called bisulfite conversion, is the basis of bisulfite sequencing (BS-seq), which has been developed to profile DNA methylation systematically when coupled to next-generation sequencing [1].

Whole genome bisulfite sequencing (WGBS) is one of the most powerful tools to profile genome-wide DNA methylation, capturing most cytosines in each context, e.g., CG, CHG, and CHH, without bias [1,2]. WGBS library construction usually takes 3–5 days. Several commercial WGBS library preparation kits have been released for different applications, e.g., ultralow input or short bisulfite conversion time. These kits provide all-in-one packages that include bisulfite conversion and library construction reagents. Though convenient, the commercial kits usually come with defined indexes and reaction numbers that can lead to limitations with respect to multiplexing with other sequencing libraries and higher costs. In this protocol, we describe WGBS library construction steps and provide a flexible alternative for researchers to generate a WGBS library without library preparation kits. The presented protocol has been applied to rice [3] and *Arabidopsis* [4,5], and is assumed to be compatible with other species.

## Materials and reagents

**Biological materials**

1. Genomic DNA (gDNA) from the organism of interest; 500 ng or more gDNA is recommended for this protocol

2. Phage lambda DNA (Thermo Scientific, catalog number: SD0021)

**Reagents**

1. DNA*Zap* ^TM^ PCR DNA degradation solutions (Invitrogen, catalog number: AM9890 or equivalent); store at 4 °C

2. RNase A, DNase and protease-free (Thermo Scientific, catalog number: EN0531); store at -20 °C

3. AMPure XP beads (Beckman Coulter, catalog numbers: A63880, A63881, or A63882); store at 4 °C

4. Molecular biology–grade absolute ethanol (Fisher Scientific, catalog number: BP2818110); store at room temperature (RT)

5. UltraPure DNase/RNase-free distilled water (Invitrogen, catalog number: 10977015); store at RT

6. UltraPure 1 M Tris-HCl, pH 8.0 (Invitrogen, catalog number: 15568025); store at RT

7. Klenow Fragment 3′→5′ exonuclease (New England Biolabs, catalog number: M210S); store at -20 °C

8. T4 DNA ligase (New England Biolabs, catalog number: M0202S); store at -20 °C

9. EpiTect Fast Bisulfite Conversion kit (Qiagen, catalog number: 59802); store parts at different temperatures according to the manufacturer’s guide

10. MinElute PCR Purification kit (Qiagen, catalog number: 28004); store the spin columns at 4 °C and the remaining at RT

11. PfuTurbo Cx hotstart DNA polymerase (Agilent Technologies, catalog number: 600410); store at -20 °C

12. Deoxynucleotide (dNTP) solution mix (New England Biolabs, catalog number: N0447S); store at -20 °C

13. dATP solution (New England Biolabs, catalog number: N0440S); store at -20 °C

14. Qubit dsDNA BR Quantification Assay kit (Invitrogen, catalog number: Q32850); store the DNA standard at 4 °C and the remaining at RT; keep the dye reagent from light

15. TapeStation D1000 ScreenTape (Agilent Technologies, catalog number: 5067-5582); store at 4 °C

16. TapeStation D1000 ladder (Agilent Technologies, catalog number: 5067-5586); store at 4 °C

17. TapeStation D1000 sample buffer (Agilent Technologies, catalog number: 5067-5602); store at 4 °C

**Solutions**

1. Elution buffer (EB) (see Recipes)

2. RNaseA stock (see Recipes)

3. RNaseA reaction (see Recipes)

4. End-repair mix (see Recipes)

5. A-tailing mix (see Recipes)

6. Ligation mix (see Recipes)

7. Amplification mix (see Recipes)

8. Qubit working solution (see Recipes)

9. 80% ethanol (EtOH) (see Recipes)

**Recipes**

**1. Elution buffer (EB)**

**Table:** 

Reagent	Final concentration	Quantity or volume
UltraPure 1 M Tris-HCl, pH 8.0	10 mM	10 μL
UltraPure DNase/RNase-free distilled water	n/a	990 μL
Total	10 mM	1 mL

**Caution:** Store at RT.

**2. RNaseA stock**

**Table:** 

Reagent	Final concentration	Quantity or volume
RNase A, DNase and protease-free	10 mg/mL	10 mg
UltraPure DNase/RNase-free distilled water	n/a	1 mL
Total	10 mg/mL	1 mL

**Caution:** Aliquot 10 μL per tube and store at -20 °C. Thaw on ice before use. Do not freeze and thaw.

**3. RNaseA reaction**

**Table:** 

Reagent	Final concentration	Quantity or volume
RNase A stock	100 μg/mL	0.5 μL
gDNA solution	n/a	n/a
UltraPure DNase/RNase-free distilled water	n/a	n/a
Total	100 μg/mL	50 μL

**Caution:** Do not re-freeze the RNAseA stock.

**4. End-repair mix**

**Table:** 

Reagent	Final concentration	Quantity or volume
Sheared DNA	n/a	30 μL
NEBNext end repair reaction buffer (10×)	1×	5 μL
NEBNext end repair enzyme mix	n/a	2.5 μL
UltraPure DNase/RNase-free distilled water	n/a	12.5 μL
Total	n/a	50 μL

**5. A-tailing mix**

**Table:** 

Reagent	Final concentration	Quantity or volume
End-repaired DNA	n/a	30 μL
10× NEB buffer 2	1×	5 μL
1mM dATP	0.2 mM	10 μL
3′→5′ Klenow exonuclease	n/a	3 μL
UltraPure DNase/RNase-free distilled water	n/a	2 μL
Total	n/a	50 μL

**6. Ligation mix**

**Table:** 

Reagent	Final concentration	Quantity or volume
10× T4 DNA ligation buffer	1×	3 μL
Adapter	2 μM	6 μL
A-tailed DNA	n/a	20 μL
T4 DNA Ligase	n/a	1 μL
Total	n/a	30 μL

**7. Amplification mix**

**Table:** 

Reagent	Final concentration	Quantity or volume
Forward primer	0.5 μM	2.5 μL
Reverse primer	0.5 μM	2.5 μL
10mM dNTP	0.2 mM	1 μL
10× Pfu Turbo Cx reaction buffer	1×	5 μL
Pfu Turbo Cx hotstart DNA polymerase	n/a	1 μL
UltraPure DNase/RNase-free distilled water	n/a	22 μL
Bisulfite converted DNA	n/a	16 μL
Total	n/a	50 μL

**8. Qubit working solution**

**Table:** 

Reagent	Final concentration	Quantity or volume
Qubit reagent	n/a	1 μL
Qubit buffer	n/a	199 μL
Total	n/a	200 μL

**9. 80% ethanol (EtOH)**

**Table:** 

Reagent	Final concentration	Quantity or volume
Absolute ethanol	n/a	4 mL
UltraPure DNase/RNase-free distilled water	n/a	1 mL
Total	n/a	5 mL

**Laboratory supplies**

1. Low-retention barrier tips (Neptune, catalog numbers: 63300746, 63300757, 63300759, and 63300749, or equivalent)

2. microTUBE AFA fiber pre-slit snap-cap 6 × 16 mm (Covaris, catalog number: 520045)

3. 1.5 mL EpiTube (Eppendorf, catalog number: 022431021)

4. PCR strips (USA Scientific, catalog number: 1402-4700, or equivalent)

5. Qubit assay tube (Axygen, catalog number: PCR-05-C)

6. TapeStation tips (Agilent Technologies, catalog number: 5067-5099)

7. TapeStation tube strips (Agilent Technologies, catalog number: 401428)

8. TapeStation tube strip caps (Agilent Technologies, catalog number: 401425)

## Equipment

1. Ultrasonicator (Covaris, model: S220)

2. TapeStation (Agilent Technologies, model: 4200, or equivalent)

3. Qubit fluorometer (Invitrogen, model: 4.0, or equivalent)

4. Thermocycler (Bio-Rad, model: T100, or equivalent)

5. Microcentrifuge (Eppendorf, model: 5425, or equivalent)

6. ThermoMixer (Eppendorf, model: R, or equivalent)

7. Vortexer (Scientific Industries, model: Vortex-Genie 2, or equivalent)

8. DynaMag2 (Invitrogen, catalog number: 12321D)

9. DynaMag-PCR magnet (Invitrogen, catalog number: 492025)

10. MS3 vortexer (IKA, catalog number: 0003617000)

11. NanoDrop (Thermo Scientific, model: ND-1000, or equivalent)

## Procedure

*Note: This protocol starts with extracted genomic DNA (gDNA). The gDNA extraction method varies in several aspects, such as organism and tissue type. Some commercial kits, i.e., Qiagen QIAAmp series, include RNaseA treatment; in that case, you can skip Section A.*

**Before you start:**

1. Wipe the benchtop with DNA*Zap* ^TM^ PCR DNA degradation solutions using clean paper towels and wait until dry.

2. Fill up an ice bucket.

3. Synthesize the following oligos (Table 1):

**Table 1.** Oligo nucleotides required for this protocol

Name	Oligo sequence	Modification	Concentration
Adapter TOP	ACACTCTTTCCCTACACGACGCTCTTCCGATCT	methylated	20 μM
Adapter BOTTOM	GATCGGAAGAGCACACGTCTGAACTCCAGTCAC	methylated 5'-phosphorylation	20 μM
Forward primer	AATGATACGGCGACCACCGAGATCTACACTCTTTCCCTACACGACGCTCTTCCGATCT		10 μM
Reverse primer	GATCGGAAGAGCACACGTCTGAACTCCAGTCACATCACGATCTCGTATGCCGTCTTCTGCTTG		10 μM

*Notes:*

*1. We recommend the Adapter TOP and BOTTOM to be PAGE-purified and the primers to be HPLC-purified.*

*2. The 5′-phosphorylation of Adapter BOTTOM is to enable the ligation with the end-repaired gDNA later in this protocol.*

*3. The underlined sequence represents the index. illumina provides 12 TruSeq 6 bp indices for multiplexing and there are other commercialized 8 bp indices released. Replace the sequence when needed.*

*4. Reconstitute and aliquot upon receiving the oligos and store at -20 °C. Avoid freeze and thaw.*

4. Anneal adapters

a. Mix 10 μL of Adapter TOP and 10 μL of Adapter BOTTOM.

b. Set up the thermocycler program as below with a lid temperature of 100 °C:

95 °C 2 min

25 °C 1 min

4 °C Hold

*Note: Set the cooling ramp rate from 95 °C to 25 °C at -0.1 °C/s.*

c. Aliquot to 10 μL per vial and store at -20 °C.

*Note: Avoid freeze and thaw.*

**A. RNaseA treatment of genomic DNA (gDNA)**

*Note: Proceed to section B if gDNA was treated with RNaseA during extraction.*

1. Heat the thermomixer to 37 °C.

2. Take out AMPure XP beads from the fridge and let them sit at RT for at least 30 min.

3. Make fresh 80% EtOH (see Recipe 9).

4. Treat the gDNA solution with 100 μg/mL RNase A for 1 h at 37 °C.

5. Purify gDNA with 1.2× volumes of AMPure XP beads (see General note 3).

a. Add ddH_2_O to the RNAse A reaction to a final volume of 100 μL.

b. Thoroughly invert the AMPure XP beads 10 times.

c. Add 120 μL of the AMPure XP beads to the reaction and pipette 5 times.

d. Quickly spin down the mixture and incubate at room temperature for 5 min.

e. Place the mixture on the magnetic stand for 5 min.

f. With the tube on the magnetic stand, remove the supernatant without disturbing the beads.

g. With the tube on the magnetic stand, add 500 μL of 80% EtOH without disturbing the beads and incubate at room temperature for 1 min.

h. Remove the EtOH.

i. Repeat steps A5g–h one more time for a total of two washes.

j. With the tube on the magnetic stand, air-dry the beads for 10 min.

k. Remove the tube from the stand, resuspend the beads with 32 μL of EB buffer, and incubate for 5 min at room temperature.

l. Place the mixture on the magnetic stand for 5 min and transfer the supernatant into a new tube.

6. Access DNA purity using the Nanodrop.

*Note: The A_260/280_ should be within the 1.8–2.0 range. The A_260/230_ should be within the range of 2.0–2.4.*

7. Measure the DNA with the Qubit dsDNA BR kit and a Qubit fluorometer.

*Note: The concentration should be greater than 12 ng/μL.*

**Pause point:** Continue to DNA shearing or store at -20 °C.

**B. gDNA shearing**

1. Thaw gDNA on ice if it is frozen.

2. Spike in 0.01% lambda phage DNA.

3. Add ddH_2_O to a final volume of 50 μL.

4. Prepare the Covaris instrument 2 h before using.

a. Fill the water tank with MilliQ water.

b. Turn on both the Covaris instrument and the water tank and set the water tank temperature to 4 °C.

i. When the water tank reaches 4 °C, open up the software and start “degas” for at least 30 min.

ii. Transfer the gDNA to a Covaris tube.

iii. Place the Covaris tube onto the Covaris holder and mount the holder to the machine with the Covaris tube below the water surface.

iv. Set up the program:

Duty cycle intensity 10%

Cycles per burst 200

Number of cycles 6

Time per cycle 1 min

v. Start the program.

vi. Transfer the sheared gDNA into an EpiTube and put on ice.

5. Proceed to Section C.

**C. End-repair**

1. Prepare the end-repair mix (see Recipe 4).

2. Set up the thermocycler with a lid temperature of 30 °C:

20 °C 30 min

4 °C Hold

3. Purify gDNA with 1.5× volumes of AMPure XP beads (see General note 3) and transfer the purified DNA into a PCR tube.

a. Add 75 μL of the AMPure XP beads to the reaction and pipette 5 times.

b. Quickly spin down the mixture and incubate at RT for 5 min.

c. Place the mixture on the DynaMag-PCR magnet for 5 min.

d. With the tube on the DynaMag-PCR magnet, remove the supernatant without disturbing the beads.

e. With the tube on the DynaMag-PCR magnet, add 200 μL of 80% EtOH without disturbing the beads and incubate at room temperature for 1 min.

f. Remove the 80% EtOH.

g. Repeat steps C3e–f one more time for a total of two washes.

h. With the tube on the DynaMag-PCR magnet, air-dry the beads for 5–10 min.

**Critical:** Do not over-dry. Elute before the bead pellet cracks.

i. Remove the tube from the stand, resuspend the beads with 30 μL of EB buffer, and incubate for 5 min at room temperature.

j. Place the mixture on the DynaMag-PCR magnet for 5 min.

k. Transfer the supernatant into a new PCR tube.

4. Proceed to Section D.

**D. A-tailing**

1. Prepare the A-tailing mix (see Recipe 5)

2. Set up the thermocycler with a lid temperature of 40 °C:

37 °C 30 min

4 °C Hold

3. Purify gDNA with 1.5× volumes of AMPure XP beads (see General note 3) and transfer the purified DNA into a PCR tube.

a. Add 75 μL of the AMPure XP beads to the reaction and pipette 5 times.

b. Quickly spin down the mixture and incubate at RT for 5 min.

c. Place the mixture on the DynaMag-PCR magnet for 5 min.

d. With the tube on the DynaMag-PCR magnet, remove the supernatant without disturbing the beads.

e. With the tube on the DynaMag-PCR magnet, add 200 μL of 80% EtOH without disturbing the beads and incubate at room temperature for 1 min.

f. Remove the 80% EtOH.

g. Repeat steps D3e–f one more time for a total of two washes.

h. With the tube on the DynaMag-PCR magnet, air dry the beads for 5–10 min.

**Critical:** Do not over-dry. Elute before the bead pellet cracks.

i. Remove the tube from the stand, resuspend the beads with 20 μL of EB buffer, and incubate for 5 min at room temperature.

j. Place the mixture on the DynaMag-PCR magnet for 5 min.

k. Transfer the supernatant into a new PCR tube.

4. Proceed to Section E.

**E. Adapter ligation**

1. Prepare the ligation mix (see Recipe 6).

2. Incubate at 16 °C overnight in a thermocycler with a lid temperature of 25 °C.

3. Purified gDNA with 1.2× volumes of AMPure XP beads (see General note 3).

a. Add 20 μL of ddH_2_O to the ligation mix for a total of 50 μL.

b. Add 60 μL of the AMPure XP beads to the ligation mix and pipette 5 times.

c. Quickly spin down the mixture and incubate at RT for 5 min.

d. Place the mixture on the DynaMag-PCR magnet for 5 min.

e. With the tube on the DynaMag-PCR magnet, remove the supernatant without disturbing the beads.

f. With the tube on the DynaMag-PCR magnet, add 200 μL of 80% EtOH without disturbing the beads and incubate at room temperature for 1 min.

g. Remove the 80% EtOH.

h. Repeat steps E3f–g one more time for a total of two washes.

i. With the tube on the DynaMag-PCR magnet, air dry the beads for 5–10 min.

**Critical:** Do not over-dry. Elute before the bead pellet cracks.

j. Remove the PCR tubes from the stand, resuspend the beads with 40 μL of EB buffer, and incubate for 5 min at RT.

k. Place the mixture on the DynaMag-PCR magnet for 5 min.

l. Transfer the supernatant to a new PCR tube.

**Pause point:** Continue to bisulfite conversion or store at -20 °C.

**F. Bisulfite conversion**

*Note: This step could be achieved by multiple commercial kits. Here, we demonstrate the EpiTect Fast Bisulfite Conversion kit. Before starting, make sure all the reagents in the kit are ready to use.*

1. Heat the thermomixer to 60 °C.

2. Add 15 μL of DNA protect buffer and 85 μL of a bisulfite solution to each tube to a final volume of 140 μL, mix thoroughly, and spin down shortly.

3. Set up the thermocycler program as follows with a lid temperature of 100 °C:

95 °C 5 min

60 °C 20 min

95 °C 5 min

60 °C 20 min

20 °C Hold

4. Briefly centrifuge the PCR tubes containing the bisulfite reaction and transfer to clean 1.5 mL EpiTubes.

5. For each tube, add 310 μL of freshly prepared buffer BL (provided with the kit) containing 10 μg/mL carrier RNA (provided with the kit).

6. Mix thoroughly and spin down briefly.

7. Add 250 μL of absolute EtOH, mix thoroughly, and then spin down briefly.

8. Transfer the solution to MinElute DNA spin columns with collection tubes (provided with the kit).

9. Centrifuge at 16,000× *g* for 1 min and discard the flowthrough.

10. Wash by adding 500 μL of buffer BW (provided with the kit) to each spin column.

11. Centrifuge at 16,000× *g* for 1 min and discard the flowthrough.

12. Add 500 μL of buffer BD (provided with the kit) to each spin column.

13. Incubate for 15 min at RT with the lid closed.

14. Centrifuge at 16,000× *g* for 1 min and discard the flowthrough.

15. Repeat the washing step (F10–11) twice for a total of 3 washes.

16. Add 250 μL of absolute EtOH to each spin column and centrifuge at 16,000× *g* for 1 min.

17. Place each spin column into a clean 1.5 mL EpiTube and open the lid.

18. Incubate on the 60 °C thermomixer to evaporate residual liquid.

19. Elute DNA by adding 16 μL of buffer EB onto the center of the spin column membrane, close the lid, and incubate for 1 min.

20. Centrifuge at 16,000× *g* for 1 min.

21. Transfer the elution to a new PCR tube.

22. Proceed to Section G.

**G. Library amplification**

1. Thaw the dNTP and 10× Pfu turbo Cx buffer on ice.

2. Prepare the library amplification mix (see Recipe 7).

3. Mix thoroughly and spin down briefly.

4. Set up the thermocycler program as follows with a lid temperature of 100 °C.

1 95 °C 2 min

2 95 °C 15 s

3 63 °C 15 s

4 72 °C 30 s

5 72 °C 2 min

6 4 °C Hold

*6–10 cycles between steps 2 and 4.

**Critical:** Too many PCR cycles will cause duplications.

5. Purify the PCR product with 0.8× AMPure XP beads (see General note 3).

a. Add 50 μL of ddH_2_O to the ligation mix for a total of 100 μL.

b. Add 80 μL of the AMPure XP beads to the ligation mix and pipette 5 times.

c. Quickly spin down the mixture and incubate at RT for 5 min.

d. Place the mixture on the DynaMag-PCR magnet for 5 min.

e. With the tube on the DynaMag-PCR magnet, remove the supernatant without disturbing the beads.

f. With the tube on the DynaMag-PCR magnet, add 200 μL of 80% EtOH without disturbing the beads and incubate at room temperature for 1 min.

g. Remove the 80% EtOH.

h. Repeat steps G5f–g one more time for a total of two washes.

i. With the tube on the DynaMag-PCR magnet, air dry the beads for 5–10 min.

**Critical:** Do not over-dry. Elute before the bead pellet cracks.

j. Remove the PCR tubes from the stand, resuspend the beads with 40 μL of EB buffer, and incubate for 5 min at RT.

k. Place the mixture on the DynaMag-PCR magnet for 5 min.

l. Transfer the supernatant into a new 1.5 mL EpiTube.

6. Proceed to Section H.

**H. Library quantification**

**H1. Qubit quantification**

1. Prepare Qubit working solution (see Recipes).

*Note: Prepare two more reactions than your sample number for standards.*

2. Retrieve new Axygen^®^ PCR-05-C tubes.

3. Add 190 μL of Qubit working solution to each of the tubes used for standards.

4. Add 10 μL of each Qubit standard to the appropriate tube.

5. Add 198 μL of Qubit working solution to the sample assay tubes.

6. Add 2 μL of the library to the sample assay tubes.

7. Mix the Qubit standards and assay tubes by vortexing.

8. Incubate at RT for 2 min.

9. Read standards and samples using Qubit fluorometer.

**H2. TapeStation quantification**

**Figure 1.**
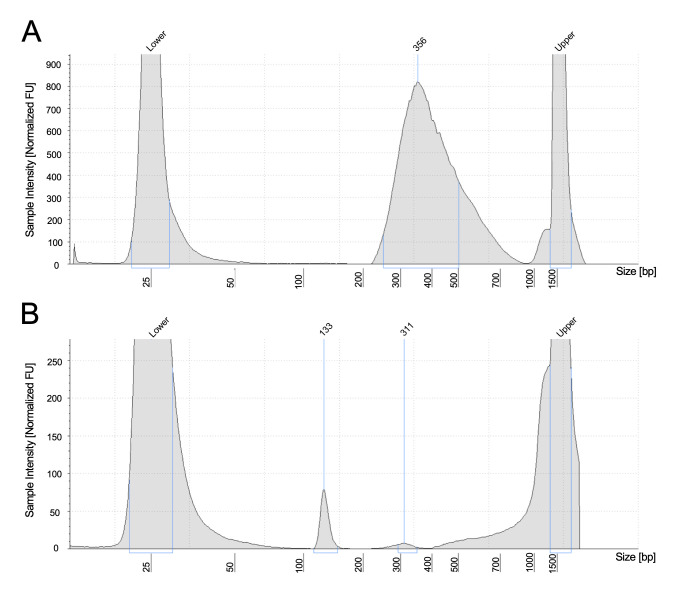
Example of TapeStation profiles. (A) The ideal library size, together with the adapter, is around 300–400 bp. (B) A failed profile shows only an adapter dimer around 130 bp.

1. Add 1 μL of DNA ladder to 3 μL of D1000 sample buffer in a tube strip, position A1.

2. Add 1 μL of library to 3 μL of D1000 sample buffer in a tube strip.

3. Mix liquids using the IKA MS3 vortexer at 2,000 rpm for 1 min.

4. Spin down for 1 min.

5. Load samples into the TapeStation instrument. Place the ladder in position A1 on the tube strip holder.

6. Remove caps of tube strips. Confirm that the liquid is positioned at the bottom.

7. Start the assay.

8. The ideal library size distribution is shown in Figure 1A. A failed library amplification shows a peak of adapter dimer.

## Validation of protocol

This protocol or parts of it has been used and validated in the following research articles:

Hsu et al. [3]. Dynamics of the Methylome and Transcriptome during the Regeneration of Rice. *Epigenomes*.

Yen et al. [4]. Deubiquitinating enzyme OTU5 contributes to DNA methylation patterns and is critical for phosphate nutrition signals. *Plant physiology*.

Hou et al. [5]. Global impacts of chromosomal imbalance on gene expression in Arabidopsis and other taxa. *PNAS*.

## General notes and troubleshooting

**General notes**

1. This protocol could take from 3 to 5 days. Plan the pauses before every experiment.

2. Arrange the equipment usage with your lab colleagues.

3. If processing more than 16 samples in a batch, while doing bead cleanup, avoid over-drying by drying 16 samples in one group while leaving the rest in 80% EtOH.

**Troubleshooting**

**Problem 1:** TapeStation profile shows a peak around 121 bp.

Possible cause: Adapter dimer.

Solution: To recover the adapter-dimer contaminated library, an additional AMPure XP bead purification could be performed with a bead to DNA ratio of 0.8×.

**Problem 2:** Low library yield.

Possible cause: Low gDNA input.

Solution: Increase input gDNA or perform additional library amplification.

**Problem 3:** Incomplete bisulfite conversion (bisulfite unconversion rate is greater than 0.1%).

Possible cause: Low DNA input quality, degraded reagents, or improper reaction temperature.

Solution: Always spike-in lambda DNA as a control. Ensure all bisulfite conversion reagents are within their expiration dates. Quantify DNA using Qubit before bisulfite conversion.

**Problem 4:** PCR inhibition from contaminants.

Possible cause: Low DNA quality.

Solution: Additional beads or column-based DNA cleanup.

**Problem 5:** High duplication rate (greater than 15%).

Possible cause: Low input gDNA quantity and over-amplified PCR cycles.

Solution: Quantify the DNA input carefully. Start with 6–8 cycles of PCR amplification, quantify, and decide if additional cycles are needed.
